# Regeneration of the Exocrine Pancreas Is Delayed in Telomere-Dysfunctional Mice

**DOI:** 10.1371/journal.pone.0017122

**Published:** 2011-02-22

**Authors:** Guido von Figura, Martin Wagner, Kodandaramireddy Nalapareddy, Daniel Hartmann, Alexander Kleger, Luis Miguel Guachalla, Harshvardhan Rolyan, Guido Adler, Karl Lenhard Rudolph

**Affiliations:** 1 Institute of Molecular Medicine and Max-Planck-Research-Group on Stem Cell Aging, University of Ulm, Ulm, Germany; 2 Department of Internal Medicine I, University of Ulm, Ulm, Germany; 3 Department of Surgery, Technical University of Munich, Munich, Germany; Chinese University of Hong Kong, Hong Kong

## Abstract

**Introduction:**

Telomere shortening is a cell-intrinsic mechanism that limits cell proliferation by induction of DNA damage responses resulting either in apoptosis or cellular senescence. Shortening of telomeres has been shown to occur during human aging and in chronic diseases that accelerate cell turnover, such as chronic hepatitis. Telomere shortening can limit organ homeostasis and regeneration in response to injury. Whether the same holds true for pancreas regeneration in response to injury is not known.

**Methods:**

In the present study, pancreatic regeneration after acute cerulein-induced pancreatitis was studied in late generation telomerase knockout mice with short telomeres compared to telomerase wild-type mice with long telomeres.

**Results:**

Late generation telomerase knockout mice exhibited impaired exocrine pancreatic regeneration after acute pancreatitis as seen by persistence of metaplastic acinar cells and markedly reduced proliferation. The expression levels of p53 and p21 were not significantly increased in regenerating pancreas of late generation telomerase knockout mice compared to wild-type mice.

**Conclusion:**

Our results indicate that pancreatic regeneration is limited in the context of telomere dysfunction without evidence for p53 checkpoint activation.

## Introduction

Telomeres represent tandem repeat sequences at the end of the chromosomes that protect chromosomes against DNA degradation, fusions, and induction of chromosomal instability. Telomeres shorten with each round of cell division due to the ‘end-replication-problem’ of the DNA polymerase [1]. Upon reaching a critical length, telomere dysfunction induces an activation of the p53-checkpoint, which, in turn, results in either apoptosis or p21-dependent cellular senescence [2]–[5]. The enzyme telomerase is able to prevent telomere shortening by de-novo synthesis of the telomere sequence. In humans, telomerase is active in germ cells, during embryogenesis, and, to some extent, in adult stem cells.

Impaired organ maintenance and regeneration are hallmarks of aging. On the molecular level, telomere shortening occurs in most human tissues during aging [6]. To what extent it causally contributes to aging in humans is still under debate. In addition, telomere shortening has been linked to various chronic diseases, such as anemia, chronic infections, Alzheimer's disease, and chronic liver disease [6]. Mutations in telomerase are the cause of dyskeratosis congenita and have also been found in pulmonary fibrosis and aplastic anemia patients [7], [8]. Telomere shortening has also been linked to the progression of chronic liver disease, especially with the formation of cirrhosis in response to chronic hepatitis [9]. Taken together, these findings indicate that telomere shortening is involved in the pathogenesis of chronic diseases and may limit organ regeneration.

In order to analyze the consequences of telomere dysfunction *in vivo*, we made use of the telomerase knockout (mTerc^−/−^) mouse [10], [11]. mTerc^−/−^ mice are lacking the RNA component of telomerase, which leads to an abrogation of the ability to elongate telomeres during cell division. These mice are characterized by continuous telomere shortening with successive generations. As a consequence, late generation mTerc^−/−^ mice show a progeroid phenotype with impaired maintenance of highly proliferative organs, such as the intestinal epithelium and the hematopoetic system [11]. In addition, studies on mTerc^−/−^ mice have revealed that telomere shortening limits liver regeneration upon acute or chronic injury [12], [13].

It is currently unknown whether telomere shortening would impair the regenerative capacity of the pancreas. Pancreas regeneration itself is crucial in response to acute or chronic pancreatitis since both processes damage tissue integrity. It has been shown that acute pancreatitis has a more unfavourable outcome in aged compared to young patients [14]. In addition, it is known that successive telomere shortening occurs in the human pancreas during aging [15]. However, the effect of age and the potential contributions of telomere shortening to pancreatic regeneration after acute pancreatitis have not yet been investigated.

In the present study, we used a late generation telomerase knockout mouse model (G3 mTerc^−/−^) in order to study consequences of telomere shortening on pancreas regeneration after cerulein-induced acute pancreatitis. In this model, repetitive cerulein injections lead to an acute inflammation with significant damage predominantly to the exocrine pancreas followed by successive complete regeneration [16]. This study provides the first experimental evidence that telomere shortening impairs regeneration of the exocrine pancreas.

## Results

### Normal pancreas development of telomerase knockout mice despite telomere shortening

**Figure 1:**
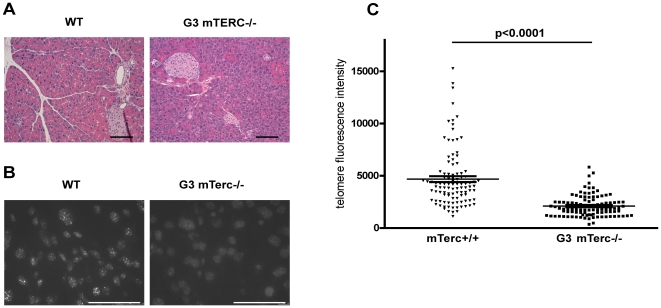
Normally developed pancreas despite shortened telomeres in telomerase knockout mice. (A) Representative H&E sections of mTerc^+/+^ and G3 mTerc^−/−^ mice reveal no differences in pancreas histology (size bar  = 1 mm). (B) Telomere qFISH analysis reveals significantly decreased telomere fluorescence intensity in pancreata of G3 mTerc^−/−^ compared to mTerc^+/+^ mice (size bar  = 50 µm). Depicted is an overlay of a representative DAPI (dark grey shaped nuclei) and corresponding telomere FISH signal (white dots; TFI  =  telomere fluorescence intensity). (C) Quantification of telomere fluorescence intensity. n = 5 mice per group; 20 nuclei per mouse were counted.

Aged telomerase knockout mice are characterized by an impairment in the maintenance of highly proliferative organs leading to intestine and bone marrow failure [11]. An analysis of histological sections revealed no morphologic abnormalities of pancreata of 8–10 month old G3 mTerc^−/−^ mice compared to mTerc^+/+^ mice (n = 75 mice per group, Figure 1A). In addition, we could not observe any change in pancreatic wet weight (7.615±0.5554 mg/g for mTerc+/+, 7.544±0.7685 mg/g for G3 mTerc^−/−^ mice, p = 0.94, n = 6 each).

Despite normal appearing pancreatic histology, quantitative fluorescence in situ hybridisation (qFISH) revealed a marked reduction in telomere fluorescence intensity (TFI) in pancreata of G3 mTerc^−/−^ mice (mean TFI: 2,103±102) compared to mTerc^+/+^ mice (mean TFI: 4,683±277, p<0.0001, Figure 1B,C). Reductions in TFI are an established measure of telomere shortening [17].

### Acute phase of cerulein-induced pancreatitis of telomerase knockout mice is similar to mTerc+/+ mice

**Figure 2:**
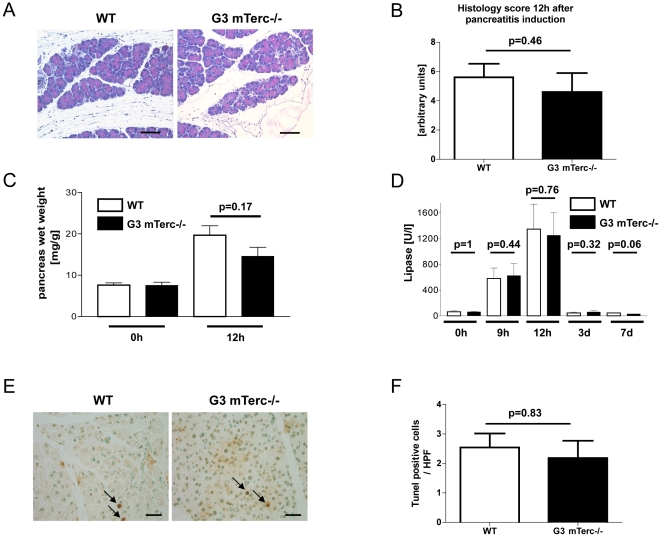
Acute pancreatitis in telomere-dysfunctional mice. (A) Representative H&E sections of indicated genotypes 12 h after pancreatitis induction (size bar  = 500 µm). (B) Histological score of the indicated genotypes at 12 h after pancreatitis induction, n = 5 per group. The histological score is based on pancreatic edema, immune cell infiltration, and necrosis. (C) Pancreatic wet weight of mTerc^+/+^ and G3 mTerc^−/−^ mice at the indicated time points after pancreatitis induction. (D) Lipase serum levels of mTerc^+/+^ and G3 mTerc^−/−^ mice at the indicated time points after pancreatitis induction. (E) TUNEL staining of pancreata of indicated genotypes 12 h after pancreatitis induction (apoptotic cells are stained dark brown and marked with a black arrow; size bar  = 200 µM) and its quantification (F), n = 5 per group. Error bars represent standard error mean (SEM).

It is not known, whether acute inflammatory conditions are altered in the context of telomere dysfunction. The peak stage of cerulein-induced pancreatitis occurs 9–12 h after the first cerulein injection. At this time point, the histological appearance of cerulein-induced pancreatitis (Figure 2A) and the histological scoring of pancreatits-associated inflammatory infiltrates, pancreatic edema, and necrosis (Figure 2B and Figure S1) revealed no difference in pancreata of 8-month-old G3 mTerc^−/−^ mice compared to mTerc^+/+^ mice (n = 5 mice per group). Similarly, the pancreatic wet weight, a measurement for pancreatits-induced edema, did not show significant differences between the two cohorts (Figure 2C). Moreover, lipase serum levels (a marker for acinar cell necrosis) did not reveal significant differences in cerulein-induced pancreatitis between the two cohorts (Figure 2D). TUNEL staining revealed similar rates of apoptosis in the pancreata of G3 mTerc^−/−^ and mTerc^+/+^ mice at the peak stage of cerulein-induced pancreatitis (Figure 2E,F). Together, these results indicated that telomere shortening did not change the severity of acute pancreatitis induced by repeated cerulein injection.

### Regeneration after acute pancreatitis is impaired in telomerase knockout mice

**Figure 3:**
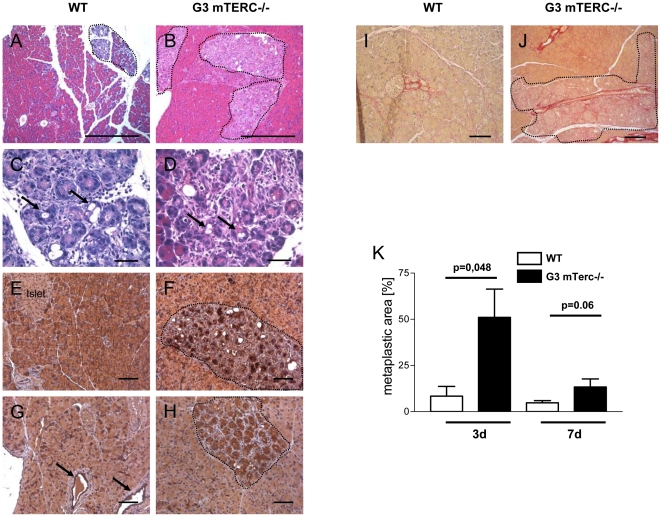
Impaired regeneration after acute pancreatitis in telomere-dysfunctional mice. Regeneration 3 d after induction of acute cerulein pancreatitis in mTerc^+/+^ (A,C) and G3 mTerc^−/−^ mice (B,D); arrows point to tubular complexes, degenerated area is encircled, H&E staining (A,B size bar  = 1 mm; C,D size bar  = 200 µm). De-differentiated tissue stains negative for amylase in G3 mTerc^−/−^ mice (F) compared to mTerc^+/+^ mice (E) with tubular complexes positive for CK-19 in mTerc^+/+^ (G; arrows point to normal ducts) and G3 mTerc^−/−^ mice (H; tubular complexes are in encircled area) (E-H size bar  = 200 µm; amylase and CK-19 were stained with NovaRed (Vectorlabs) represented by red-brown color). Sirius red staining (stains fibrotic tissue red) reveals marked fibrosis in the de-differentiated tissue 3 d after pancreatitis induction in G3 mTerc^−/−^ (J) compared to mTerc^+/+^ mice (I; I,J size bar  = 500 µm). (K) Quantification of the degenerated area in mTerc^+/+^ and G3 mTerc^−/−^ mice. Error bars represent SEM.

In order to investigate the regeneration from acute cerulein-induced pancreatitis, pancreatic tissue of mTerc^+/+^ and G3 mTerc^−/−^ mice was examined 3 days and 7 days after pancreatitis induction. In the course of acute cerulein-induced pancreatitis, pancreata of 8-month-old mTerc^+/+^ mice had almost completely regenerated after 3 days (Figure 3A,C). At this time point, age-matched G3 mTerc^−/−^ mice showed distinct areas of the exocrine pancreas with reduced eosin-positive cytoplasm and lack of apical zymogen granules. Moreover, these areas showed acinar to ductal metaplasia - a typical feature of cerulein-induced pancreatitis [18]. The metaplastic areas revealed a loss of amylase expression (Figure 3E,F and Figure S2A,B) and an expansion of ductal cells with formation of tubular complexes (Figure 3G,H and Figure S2C,D). A quantification of the amount of these metaplastic areas revealed a significant difference between the two cohorts 3 days after cerulein injection (p = 0.048; Figure 3K). In addition, Sirius red staining 3 days after pancreatitis induction revealed increased fibrosis in pancreata of G3 mTerc^−/−^ mice compared to mTerc^+/+^ mice (Figure 3I,J).

Apart from an increased occurrence of metaplastic tissue 3 days after pancreatitis induction, there were no signs of islet degeneration, evolution of diabetes mellitus, weight loss, or diarrhea upon 3-week-follow-up of G3 mTerc^−/−^ mice. Histologically, the pancreata appeared normal on H&E staining after 3 weeks (Figure S3 and data not shown).

**Figure 4:**
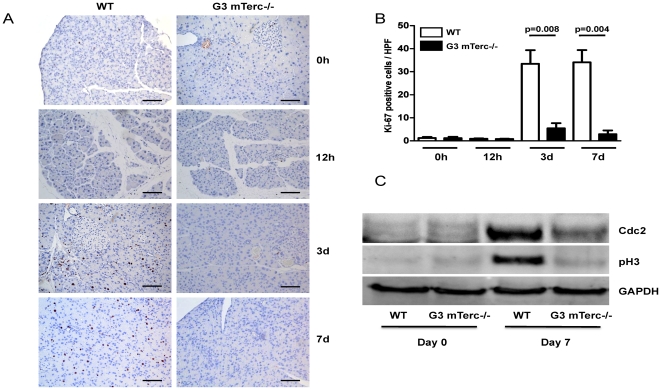
Block of proliferation in telomere-dysfunctional mice. (A) Representative pictures of Ki-67 staining of pancreata of indicated genotype and time point after pancreatitis (Ki-67 positive nuclei are stained black; size bar  = 500 µm). (B) Quantification of Ki-67 positive cells of mTerc^+/+^ and G3 mTerc^−/−^ mice at indicated time points after pancreatitis induction. (C) Analysis of cell cycle proteins in pancreata of mTerc^+/+^ and G3 mTerc^−/−^ mice before and 7 d after pancreatitis induction (pH3 =  phospho Histone 3 Serine 10).

Furthermore, proliferating acinar cells were investigated by Ki-67 staining. No difference in proliferating acinar cells could be determined in un-stimulated pancreata of both groups (1.220±0.4810 positive cells/high power field (HPF) n = 6 in mTerc+/+ and 1.175±0.5921 positive cells/HPF n = 4 in G3 mTerc^−/−^ mice, p = 0.62). In contrast, the proliferative response of acinar cell at day 3 after cerulein-induced pancreatitis was markedly reduced in G3 mTerc^−/−^ mice (5.429±2.241 Ki-67-positive cells per high power field, n = 5 mice) compared to mTerc^+/+^ mice (33.43±5.912 Ki-67 positive cells per high power field, n = 5, p = 0.008, Figure 4A,B). A similar impairment in pancreas regeneration of G3 mTerc^−/−^ mice compared to mTerc^+/+^ mice was seen at day 7 after cerulein-induced pancreatitis (Figure 4A, B). The Ki-67-positive acinar cells were evaluated in the non-metaplastic areas of the pancreas. In line with the Ki-67 analysis on cell proliferation, mTerc^+/+^ mice exhibited a stronger induction of proliferation markers characterizing the G2/M stage of the cell cycle (phospho-histone 3 (pH 3) and CDC2) compared to G3 mTerc^−/−^ mice (Figure 4C).

### DNA damage response in telomere-dysfunctional mice

**Figure 5:**
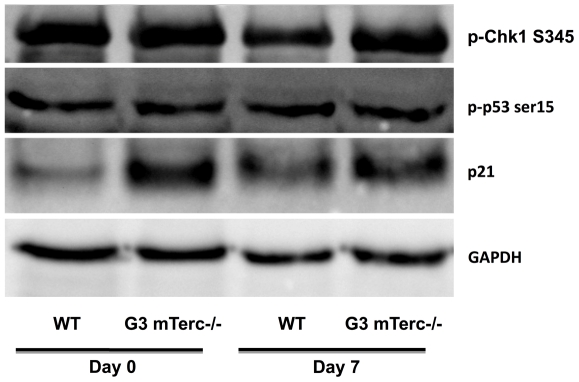
DNA damage response protein expression during regeneration of the pancreas. pChk1, p53 and p21 expression in pancreata of mTerc^+/+^ and G3 mTerc^−/−^ mice before and 7 d after pancreatitis induction.

In order to decipher possible mechanisms limiting pancreas regeneration in G3 mTerc^−/−^ mice in response to cerulein-induced pancreatitis, mediators of the DNA damage response were analyzed in resting, non-injured pancreata (day 0) and in damaged pancreata (day 7 after cerulein application). Western blot analysis revealed an increased expression of p21 in resting, non-injured pancreata of G3 mTerc^−/−^ mice compared to mTerc^+/+^ mice. However, 7 days after cerulein-induced pancreatitis, no differences in the levels of activated p53 or p21 were observed in pancreata from G3 mTerc^−/−^ mice compared to mTerc^+/+^ mice (Figure 5). Phosphorylated Chk1 (an upstream kinase inducing DNA damage responses) was strongly expressed in non-injured pancreas of G3 mTerc^−/−^ mice and mTerc^+/+^ mice. In mTerc^+/+^ mice, phospho-Chk1 expression declined during pancreas regeneration, whereas G3 mTerc^−/−^ mice failed to inactivate phospho-Chk1 in response to pancreatic injury (Figure 5).

## Discussion

The present study shows that pancreas regeneration in response to cerulein-induced pancreatitis is impaired in G3 mTerc^−/−^ mice with shortened telomeres compared to mTerc^+/+^ mice with longer telomere reserve. The experiments revealed no significant influence of telomere shortening on the severity of pancreatitis induced by cerulein injection. The severity of pancreatitis was evaluated histologically by lipase measurement and by pancreatic wet weight. In addition, TUNEL staining for evaluation of apoptosis that typically occurs in response to acute pancreatitis, was performed. Together, these data showed no changes between G3 mTerc^−/−^ compared to mTerc^+/+^ mice indicating that differences in the regenerative response were not due to differences in the initiated levels of tissue damage.

The delay in pancreatic regeneration in the context of telomere shortening was associated with the persistence of metaplastic exocrine tissue, the formation of tubular complexes, and an increase in fibrotic tissue at 3 days after tissue injury. Increased pancreatic fibrosis and tubular complexes are also a hallmark of human chronic pancreatitis [19], [20]. Based on our study, it should be evaluated whether telomere shortening correlates with the development of fibrosis and tubular complexes in patients with chronic pancreatitis. However, up to now it is not known, whether telomere shortening can be observed in the evolution of human chronic pancreatitis.

Delayed pancreas regeneration was found to be associated with impaired proliferation rates in pancreata of cerulein-treated G3 mTerc^−/−^ mice compared to mTerc^+/+^ mice. Previous studies have revealed that proliferating cells seen after acute cerulein-induced pancreatitis are mainly differentiated acinar cells that contribute to tissue repair [21]. Studies on telomerase knockout mice have shown that telomere shortening affects stem cell compartments of highly proliferative organs and impairs regeneration of somatic tissues by activating p21-dependent cell cycle arrest [22]. In contrast, the current study did not reveal a pronounced induction of p53 or p21 in pancreata of G3 mTerc^−/−^ compared to mTerc+/+ mice. However, G3 mTerc^−/−^ mice showed an impaired inactivation of phospho-Chk1 in response to pancreas injury compared to mTerc^+/+^ mice. These data suggest that p53-independent pathways contribute to impairments in pancreas regeneration in response to telomere shortening possibly involving Chk1.

It has been shown that transient dedifferentiation and proliferation of acinar cells is required for exocrine pancreas regeneration after cerulein damage [21], [23]. It is conceivable that impaired acinar cell regeneration led to delayed pancreas regeneration and a prolonged persistence of metaplastic tissue in the pancreata of G3 mTerc^−/−^ mice compared to mTerc^+/+^ mice. However, G3 mTerc^−/−^ mice did not fail to complete pancreas regeneration, the process was just delayed. It remains to be investigated whether a delay in pancreas regeneration could lead to manifest pancreas defects in the context of chronic organ damage. If so, telomere shortening could represent a causal factor influencing disease progression in chronic pancreatitis.

In summary, this study presents the first experimental evidence that the induction of acute pancreatitis by cerulein treatment is not altered by telomere shortening in mice. However, telomere shortening was associated with delayed regeneration after acute pancreatitis apparently involving p53- and p21-independent mechanisms.

## Materials and Methods

### Animal experiments

mTerc+/+ and mTerc^−/−^ mice were held in a pathogen-free area (20–22°C) with free access to food and water.

For the induction of pancreatitis, mice were starved for 18 h and injected 6 hourly doses of 100 µg/kg cerulein (Takus, Pfizer) i.p. Mice were sacrificed at the indicated time points after the first injection of cerulein.

The animal experiments were approved by the government of the state of Baden-Württemberg (animal protocol number 35/915.81-3/919).

### Histology score

The histology score was calculated blindly by M.W. The score is based on the level of pancreatic edema, immune cell infiltrate, and acinar cell necrosis and was determined as previously described [24].

### qFISH analysis for telomere length

Telomere length was determined on 5 µm-thick paraffin sections using quantitative fluorescence in situ hybridization (qFISH) according to previously reported methods [25]. Briefly, unmasked sections were incubated in pepsine solution (200 mg pepsine, 168 µl HCL 37%, up to 200 ml H_2_O) for 6 min at 37°C. After washing with phosphate-buffered saline, slides were hybridized with hybridization mix (10 mmol/L Tris pH 7.2; MgCl2 buffer: 7.02 mmol/L Na2HPO3, 2.14 mmol/L MgCl2, 0.77 mmol/L citric acid; 70% deionized formamide; 0.5 µg/mL PNA probe 5-Cy3-CCC TAA CCC TAA CCC TAA-3 Applied Biosystems; 0.25% blocking reagent from Roche) at 80°C for 3 min followed by a 2 h incubation at RT. After washing with formamide solution (70 ml formamide, 1 ml 1 M Tris pH7,2, 1 ml BSA 10%, up to 100 ml H_2_O) and phosphate-buffered saline, sections were mounted with DAPI. The relative telomere length was determined by quantification of the fluorescence intensity using TFL software [17].

### Lipase measurement

Lipase was measured in serum of the mice in the clinical chemistry department of the University Hospital of Ulm with the same protocol and equipment as for routine measurement of human samples. Before measurement, the serum was diluted 1∶4 with PBS.

### Immunohistochemistry

H&E staining was performed according to standard procedures. Quantification of degenerated tissue was performed on serial pictures of H&E-stained pancreatic tissue (50x magnification; 10 pictures per pancreas) by determination of the degenerated area using Image J software. The degenerated area was identified by lack of eosin-positive cytoplasm and disturbed tissue integrity on H&E-stained sections. These areas were encircled and quantified using ImageJ (an example is shown in Figure 3A,B).

Immunofluorescence was performed on paraffin-embedded pancreas using primary anti-amylase (Santa Cruz) and anti-CK19 (Santa Cruz). Ki-67 immunohistochemistry was performed on paraffin-embedded pancreas using primary anti-Ki67 (Monosan). Primary and secondary antibody dilutions were 1∶200 and 1∶250, respectively. Immunohistochemistry was performed on paraffin-embedded pancreas using primary anti-amylase (Santa Cruz) and anti-CK19 (Santa Cruz). Primary and secondary antibody dilutions were 1∶100 and 1∶200, respectively. For development ABC Kit (Vector labs) and NovaRed peroxidase substrate kit (Vector labs) was used. TUNEL staining was performed according to the manufacturer's protocol (Roche). Sirius Red staining was performed according to standard procedures. Sirius red stains fibrotic tissue red. The metaplastic areas were identified on Sirius red-stained slides by disturbed tissue morphology and lack of yellow-stained cytoplasm. For amylase and CK19 staining, at least 3–5 mice per time point and per genotype were stained and representative pictures were taken. For Ki-67 staining, non-metaplastic areas of the pancreas were evaluated since the metaplastic areas are infiltrated by many immune cells that proliferate and do not represent proliferation of acinar cells. The magnification of the evaluated pictures was 200x and the Ki67-positive cells per picture was determined.

### Immunoblot analysis

Whole-cell extracts were prepared according to standard protocols and tested by western blot using anti-P21 (Santa Cruz), anti-pP53 (Cell Signalling), anti-pChk1 (Cell Signalling), anti-Cdc2 (Santa Cruz), anti-pH3 (Santa Cruz), and anti-GAPDH (Bethyl laboratory). Dilutions of the primary and secondary antibodies were 1∶1,000 1∶10,000, respectively. Western blots were performed on pooled samples of n = 3–5 pancreata per time point and genotype.

### Blood sugar measurement

Blood sugar was measured using a glucometer (OneTouch Ultra) by withdrawing blood from the tail vene of non-starved mice in the afternoon.

### Statistical analysis

Statistical analysis was performed with GraphPad Prism using rank-based Mann-Whitney test.

## Supporting Information

Figure S1 **Histological score in acute pancreatitis.** Histological score of the indicated genotypes at 12 h after pancreatitis induction, n = 5 per group. The histological score is depicted separately for (A) pancreatic edema (p = 0.55), (B) immune cell infiltration (p = 0.42), and (C) necrosis (p = 0.69).
(TIF)

Figure S2 **Metaplastic tissue after acute pancreatitis in telomere-dysfunctional mice.** Metaplastic tissue 3 d after induction of acute cerulein pancreatitis in mTerc^+/+^ and G3 mTerc^−/−^ mice was analyzed by amlylase (A,B) and CK-19 staining (C,D; A-D size bar  = 100 µm). De-differentiated tissue (encircled area) stains negative for amylase in G3 mTerc^−/−^ mice (B) compared to mTerc^+/+^ mice (A) with tubular complexes (arrows) positive for CK-19 in mTerc^+/+^ (C) and G3 mTerc^−/−^ mice (D). Fluorescence staining: amylase  =  red, DAPI  =  blue, CK-19  =  red, DAPI  =  blue.
(TIF)

Figure S3 **Blood sugar and body weights are stable after induction of acute pancreatitis.** Acute pancreatitis was induced using cerulein in mTerc^+/+^ (n = 7) and G3 mTerc^−/−^ (n = 7) mice. The indicated genotypes were followed over 3 weeks measuring body weight (A) and blood sugar (B) at the given time points.
(TIF)
