# Supplementary figures and images for: Regeneration of the Exocrine Pancreas Is Delayed in Telomere-Dysfunctional Mice
Source: PLoS One. 2011 Feb 22;6(2):e17122. doi: 10.1371/journal.pone.0017122 (PMC3043103; doi:10.1371/journal.pone.0017122)

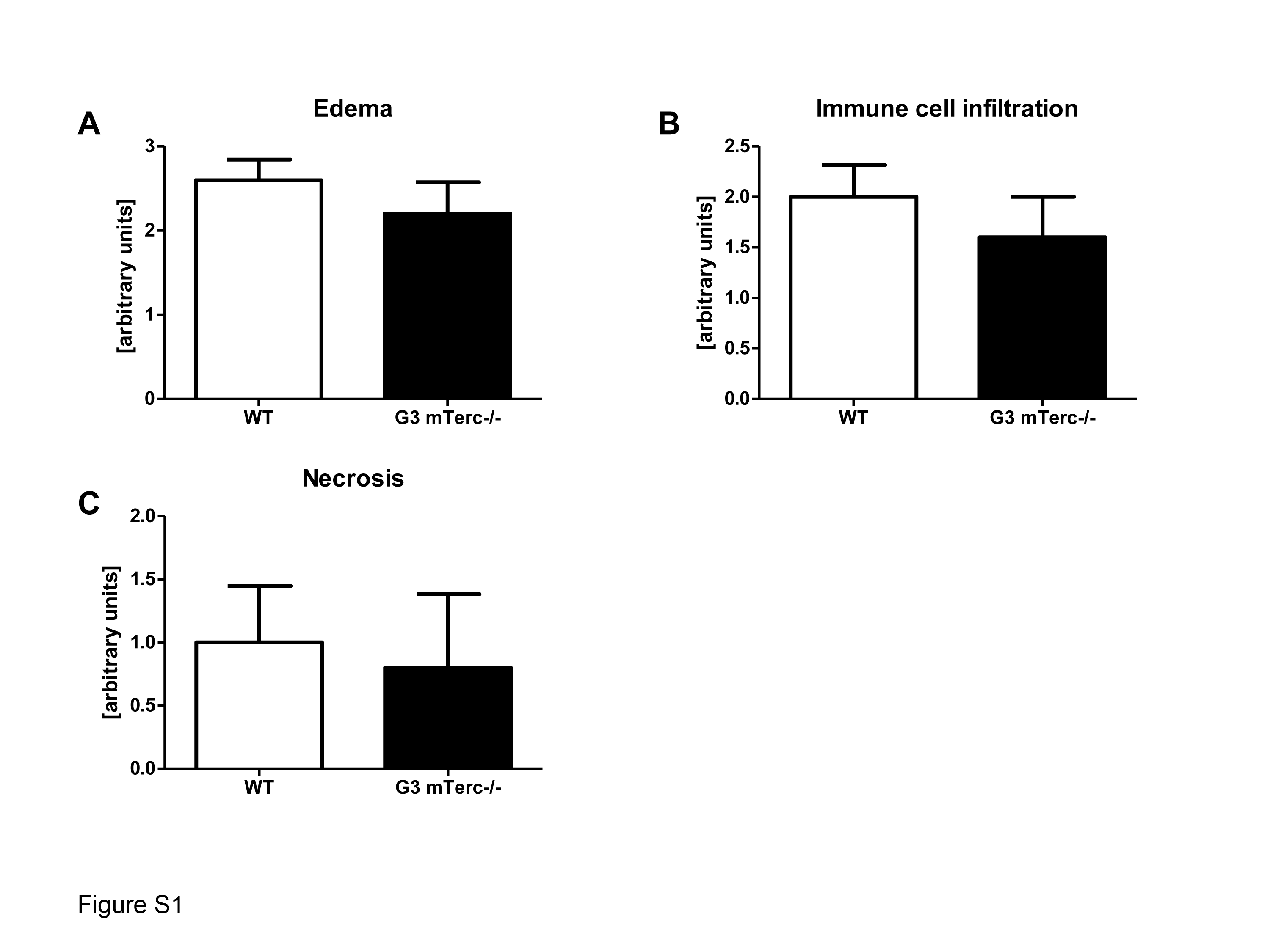

Supplement: Figure S1 — Histological score in acute pancreatitis. Histological score of the indicated genotypes at 12 h after pancreatitis induction, n = 5 per group. The histological score is depicted separately for (A) pancreatic edema (p = 0.55), (B) immune cell infiltration (p = 0.42), and (C) necrosis (p = 0.69). (TIF) [file pone.0017122.s001.tif]

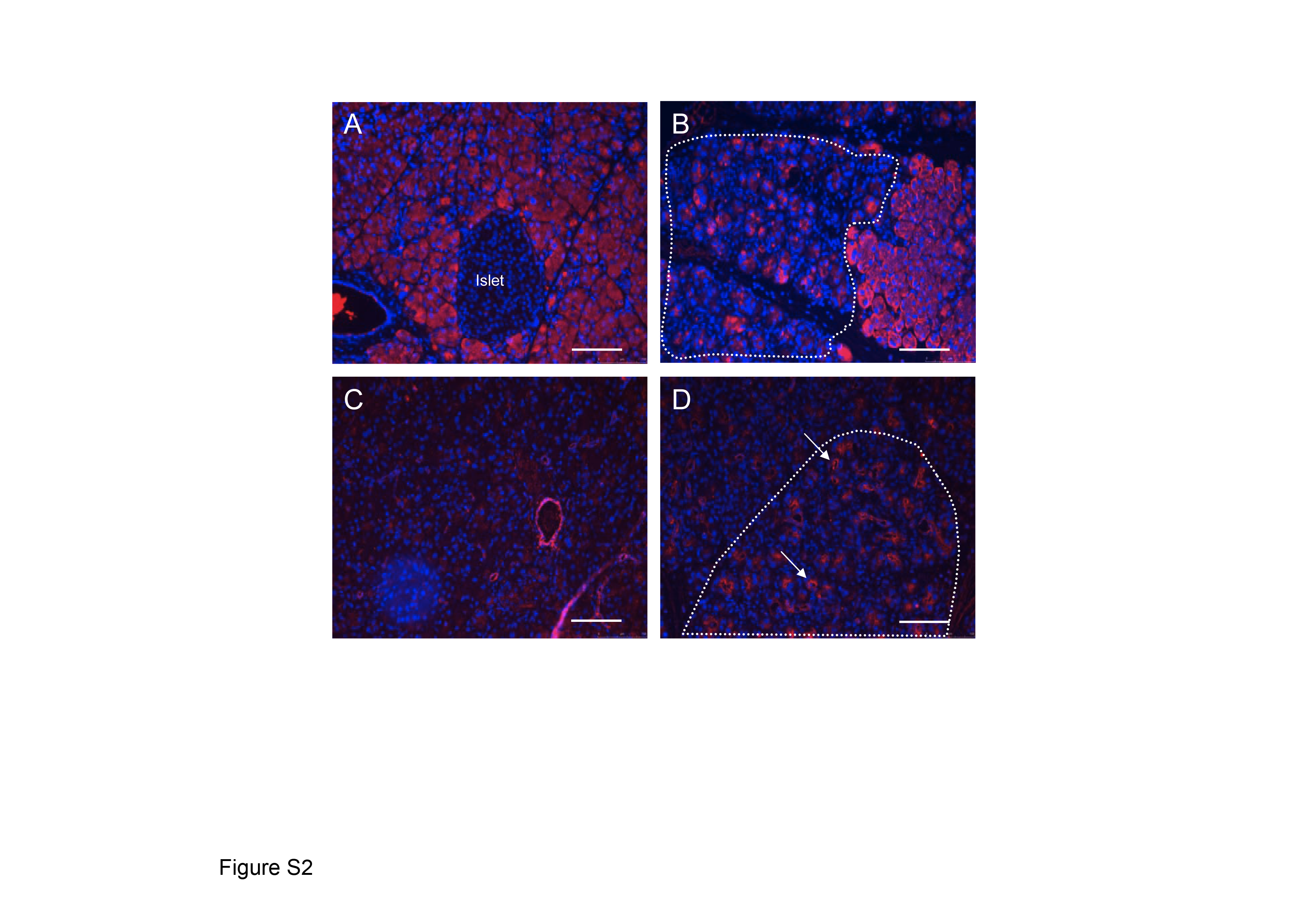

Supplement: Figure S2 — Metaplastic tissue after acute pancreatitis in telomere-dysfunctional mice. Metaplastic tissue 3 d after induction of acute cerulein pancreatitis in mTerc+/+ and G3 mTerc−/− mice was analyzed by amlylase (A,B) and CK-19 staining (C,D; A-D size bar = 100 µm). De-differentiated tissue (encircled area) stains negative for amylase in G3 mTerc−/− mice (B) compared to mTerc+/+ mice (A) with tubular complexes (arrows) positive for CK-19 in mTerc+/+ (C) and G3 mTerc−/− mice (D). Fluorescence staining: amylase = red, DAPI = blue, CK-19 = red, DAPI = blue. (TIF) [file pone.0017122.s002.tif]

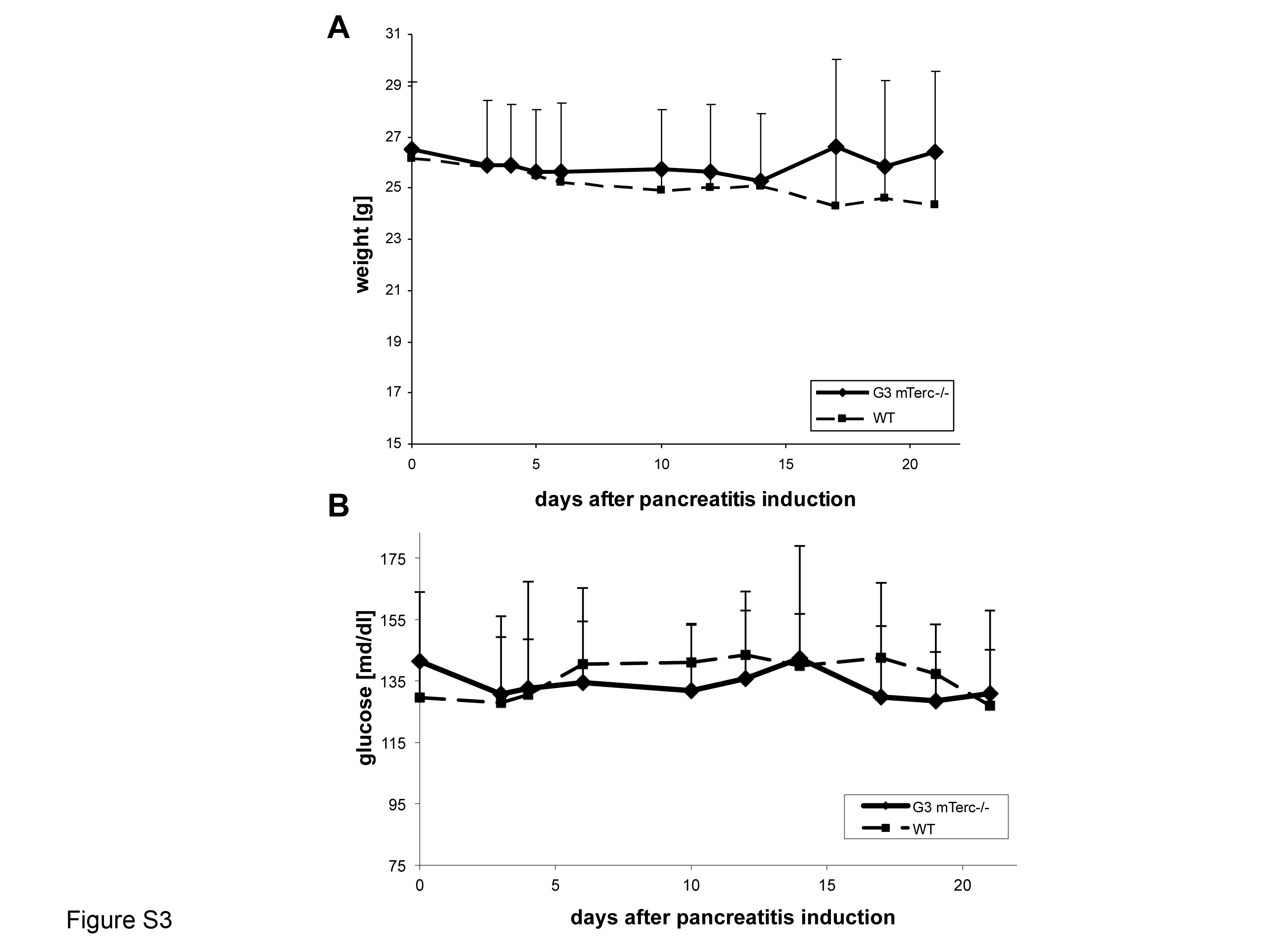

Supplement: Figure S3 — Blood sugar and body weights are stable after induction of acute pancreatitis. Acute pancreatitis was induced using cerulein in mTerc+/+ (n = 7) and G3 mTerc−/− (n = 7) mice. The indicated genotypes were followed over 3 weeks measuring body weight (A) and blood sugar (B) at the given time points. (TIF) [file pone.0017122.s003.tif]
